# Cord Blood Cells Responses to IL2, IL7 and IL15 Cytokines for mTOR Expression

**DOI:** 10.15171/apb.2017.010

**Published:** 2017-04-13

**Authors:** Anahita Mohammadian, Elahe Naderali, Seyedeh Momeneh Mohammadi, Aliakbar Movasaghpour, Behnaz Valipour, Mohammad Nouri, Hojjatollah Nozad Charoudeh

**Affiliations:** Stem Cell Research Center, Tabriz university of Medical Sciences, Tabriz, Iran.

**Keywords:** Cord blood, mTOR, Cytokines

## Abstract

***Purpose:*** Mammalian target of rapamycin (mTOR)is important in hematopoiesis and affect cell growth,differentiation and survival. Although previous studies were identified the effect of cytokines on the mononuclear cells development however the cytokines effect on mTOR in cord blood mononuclear cells was unclear. The aim of this study was to evaluate mTOR expression in cord blood mononuclear and cord blood stem cells (CD34^+^ cells) in culture conditions for lymphoid cell development.

***Methods:*** Isolation of The mononuclear cells (MNCs) from umbilical cord blood were done with use of Ficollpaque density gradient. We evaluated cultured cord blood mononuclear and CD34^+^ cells in presece of IL2, IL7 and IL15 at distinct time points during 21 days by using flow cytometry. In this study, we presented the role of IL2, IL7 and IL15 on the expression of mTOR in cord blood cells.

***Results:*** mTOR expression were increased in peresence of IL2, IL7 and IL15 in day 14 and afterword reduced. However in persence of IL2 and IL15 expression of mTOR significantly reduced. mTOR expression in CD34^+^ cells decreased significantly from day7 to day 21 in culture.

***Conclusion:*** cytokines play important role in mTOR expression during hematopoiesis and development of cord blood mononuclear cells.

## Introduction

Mammalian target of rapamycin (mTOR), a serine/threonine kinase has important role in cell growth, differentiation and survival in hematopoiesis.^1-3^ both extracellular and intracellular signals can activate mTOR complex includs mTOR complex 1(mTORC1) and mTOR complex 2(mTORC2). Every changes in cells and microenvironment of cells for example cell nutrient ,stress, cytokine , hormone receptors and immuneregulatory signals are able to activate mTOR signaling pathway.^4-6^ It has shown that mTOR pathway is clearly important in regulation of adaptive immune cells activation.^7,8^ Recently studies suggested that mTOR controls the activity of B, T and natural killer cells^4^ antigen receptors and vis versa cytokine receptors ( for example IL-2 receptor)can activate mTOR signaling.^8,9^ Morever mTOR have a critical role in the decisions between effector and regulatory T cell lineage commitment,^10^ and influences on the migratory properties of murine CD8^+^ T lymphocytes.^8,11^ Despite of limited studies about mTOR role in B cells activity, it was shown that inhibition of mTOR by rapamycin reduce B cell proliferation and differentiation of plasma cell.^4,12,13^ Cell cycle progression from G1 into S phase was controlled by mTOR in NK cells,^14^ however mTOR couldnot affect on NK cell cytotoxicity and cytokine production.^14,15^ IL-2 plays important role in T cell growth, proliferation of activated B cells and on NK cells differentiation.^16^ Also IL15 has important role in NK and CD8 T cell devlopment.^17,18^ It has shown that IL-7 is a key cytokine in B, T cell proliferation and thymic NK cell development.^16^

It is clearly known that cord blood cells are an important source for stem cell transplantation and immune cell therapy. It is well to underestand mTOR expression during development of B, T and NK cells from cord blood cells.

Herein, we evaluated mTOR expression in mononuclear and CD34^+^ umbilical cord blood cells and wethere IL-2, IL-7 and IL-15 could alter mTOR expression during in vitro culture.

## Materials and Methods

### Mononuclear cord blood isolation and CD34^+^ cells enrichment

Cord blood sampling has been done as reported in previous studies^16,19-21^ Umbilical‏ cord blood samples of full-term normal deliveries assembled and diluted 1:2 with phosphate buffered saline (PBS) plus 10% fetal bovine serum (FBS). Separating of mononuclear cells were done with use of Ficollpaque (GE healthcare -1.078 g/ml), by centrifuge. Isolated MNCs collected and washed twice in RPMI 1640(Gibco) plus 5% fetal bovine serum (FBS; Gibco). Cord blood mononuclear cells (MNCs) were incubated with 100 µl of CD34^+^ micro beads (Miltenyi Biotec, Germany Cat no: 130100453) for 30 minutes, cells were passed through LS MACS column (Miltenyi Biotec, Germany) and enriched CD34^+^ cells were collected in 15 ml tubes by flushing the column. Purity of CD34^+^ cells evaluated by flow cytometry in FACSCalibur (BD Bioscience) and data analyzed by Flow software version X.0.7.

### Culture condition

Seeding of the 10^5^ MNCs and isolated CD34+ cells were accomplished in 96-well plates in 250 µL of RPMI1640 supplemented with 20% FBS, 1% penicillin/streptomycin (Gibco), plus cytokines with final concentrations of: SCF (40 ng/ml), Flt3 ligand (FL, 40 ng/mL), interleukin-7 (IL-7, 40 ng/mL), IL-15 (40 ng/mL), and IL-2 (40 ng/mL) (all cytokines were purchased from PeproTech, USA). All cultures have done for 21 days at 37°C with replacing of half of the culture medium every week. Cultured cells were collected in indicated days and analyzed by flow cytometry for mTOR positive cells.

### Flow cytometry

Harvested cells were incubated with monoclonal mTOR Antibody (Novus Biologicals USA, Cat no: IC1537P) for 20 minutes in 4 degrees. Stained cells were evaluated by BD caliber (BDebioscience). Between 10,000 to 30,000 events were collected and analyzed using BD flowjo.

## Result

### mTOR expression in umbilical cord blood mononuclear cells during culture with by existence of Cytokines 

Mammalian target of rapamycin (mTOR) has important role in cell growth, differentiation and survival in hematopoiesis.^1-3^

**Figure 1:**
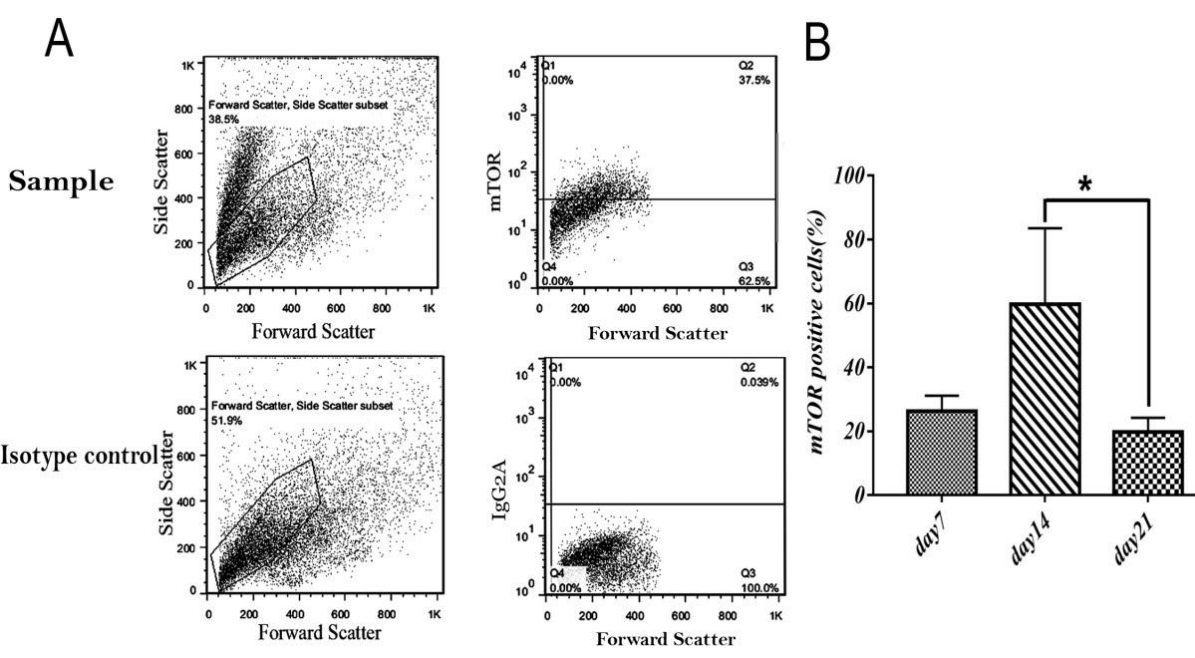
Expression of mTOR in cord blood mononuclear cells. (A) Representative FACS plots for mTOR expression. (B) Mean(SD) proportion of mTOR expression was evaluated in harvested cord blood mononuclear cells in indicated time points.in presence of SCF+FLt3+IL2+IL7+IL15. Values shown are mean ± SD from 3 independent experiments with 20-30 wells analyzed (*p <0.05).

We cultured 1x 10^5^ cord blood mononuclear cells and evaluated the relation between IL2, IL7 and IL15 cytokines and expresssion of mTOR in vitro by FACS at indicated time points (Figure 1-A).

mTOR expression in presence of all cytokines increased in day14 (59.7%) and decreased in day 21 (19.7) in compaire with day 7 ( 26.4% ). The highest mTOR expression was seen in day 14 and There was significant decline of mTOR expression in day 21 (Figure 1-B).

### Rlationship between mTOR expression and immune cell cytokines 

**Figure 2:**
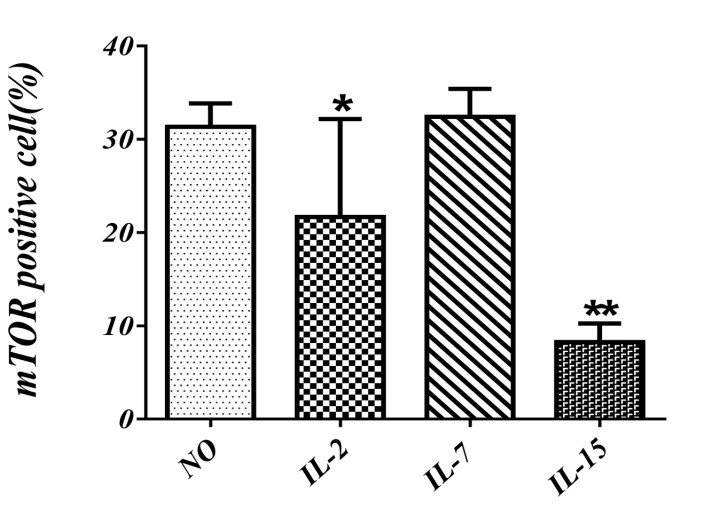
Evaluation of mTOR expression in cord blood mononuclear cells in vitro in presence of different cytokines at day 21. SCF and Flt3L have been included in all groups. Values shown are mean ± SD from 3 independent experiments with 10-12 wells analyezed in each groups ( **p <0.01 ).

To evaluate the effect of IL2, IL7 and IL15 on mTOR expression, cord blood mononuclear cells were culcured with IL2, IL7 and IL15 for 21 days. The SCF and Flt3 were suplemented in to all groups. mTOR expression was significantly lower in presence of IL15 (8.2%). Also mTOR expression reduced after co-culture with IL2 (22%) but did not altered in presence of IL7 (32.4 %) in comparison with SCF and Flt3 (31%) (Figure 2).

### mTOR expression in CD34^+^ cells

**Figure 3:**
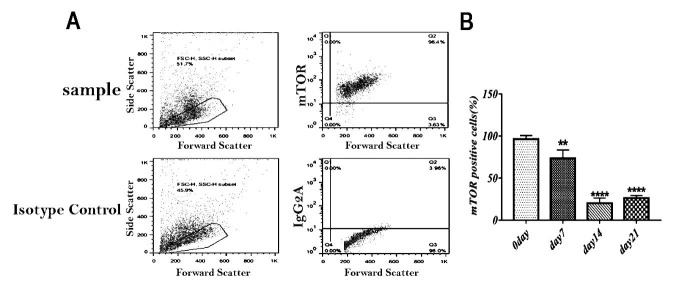
Expression of mTOR in cord blood CD34+ cells in vitro in indicated time points. (A) Representative FACS plots, (B) Mean(SD) percentage of mTOR expression in harvrested cells in different time points. Mean value preseanted from 12-18 wells were analyzed. Pvalue between day 7 and 14 and 21 ( **p <0.01, and ****p<0.0001).

We cultured 1x10^5^ CD34+ cells for 21 days without cytokines and observed expression of mTOR by flow cytometry in vitro conditions at distinct days (Figure 3-A).

the percentage of mTOR positive cells decreased from day 0 to day 21 significantly from 96% to 26%. mTOR expression was lowest in day 14 (20 %) (Figure 3-B).

## Discution

mTOR signaling is necessary during immune cell development, particularly in activated cells that are proliferative (for example activated T and B lymphocytes) and illustrated that mTOR activation in immune cells is higher than most of the other non-immune cells during development.^8,19,20^ mTOR is involve in sensing of the immune microenvironment and dictating immune function and differentiation.^6^ In this study we showed that mTOR expression in cord blood mononuclear as well as in CD34^+^ cells decreased during development which was affected by cytokinese. IL2 and IL15 had dominant role,in particular mTOR expression was influenced by IL15 more than IL2. Cytokines are soluble mediators of intercellular signals and regulate and activate the adaptive and innate immunity.^21,22^ Immune cell cytokines(IL-2, IL-7 and IL-15) control development of Natural killer cell, T and B lymphocyte and regulate hematopoiesis, proliferation, self-renewal, differentiation and senescence of HSCs (Hematopoietic stem cells).^23,24^ mTOR is sensitive to the various environmental or cellular signals and the fate of immune cells was affected by interaction of these signals on each other.^5,25-27^ mTOR plays essential role in lymphocytes.^8^ mTOR via effect on T-bet expression and IL-7 and IL-15 receptors can control STAT5 signaling indirectly,^5^ also mTOR is activated in response to IL-2 signaling. IL-2 maintains the tolerance between effector and regulatory T cells.^10^ mTOR controls proper migration of CD8^+^ T lymphocytes, although its not completely demonstrated in vivo and require more evidence. However mTOR implicate in controlling intraction between actin and microtubule cytoskeletons in T cells. Progression of cell cycle from G1 into S phase controlled by mTOR pathway in IL-2-stimulated T lymphocytes. deletion of the Cdk inhibitor protein p27Kip1 by IL-2 is able activate Cdk that rapamycin prevente this process in T cells.^28^ Also cell cycle progression was controlled by mTOR in (NK) cells.^15^

## Conclusion

Taken together, mTOR expreseed in cord blood cells cells during culture with IL2, IL7 and IL15 and it is important factor in Immune cell development in particular in cord blood derived B, T and NK cell in response to key immune cell cytokines. It is important to ivestigate mTOR behaveier in furture study.

## Acknowledgments

This work has been approved by Novin School of Advanced Medical Sciences and financially supported by Research Council of Tabriz University of Medical Sciences with Grant code: 5.104.1209.

## Ethical Issues

This study was approved by ethical committee of Tabriz University of Medical Sciences with ethical number: 5.4.10696.

## Conflict of Interest

The authors report no conflicts of interest.

## References

[1] Drayer AL, Olthof SG, Vellenga E (2006). Mammalian Target of Rapamycin Is Required for Thrombopoietin-Induced Proliferation of Megakaryocyte Progenitors. Stem Cells.

[2] Cruz R, Hedden L, Boyer D, Kharas MG, Fruman DA, Lee-Fruman KK (2005). S6 kinase 2 potentiates interleukin-3-driven cell proliferation. J Leukoc Biol.

[3] Rohrabaugh SL, Campbell TB, Hangoc G, Broxmeyer HE (2011). Ex vivo rapamycin treatment of human cord blood CD34+ cells enhances their engraftment of NSG mice. Blood Cells Mol Dis.

[4] Xu X, Ye L, Araki K, Ahmed R (2012). mTOR, linking metabolism and immunity. Semin Immunol.

[5] Saleiro D, Platanias LC (2015). Intersection of mTOR and STAT signaling in immunity. Trends Immunol.

[6] Delgoffe GM, Pollizzi KN, Waickman AT, Heikamp E, Meyers DJ, Horton MR (2011). The kinase mTOR regulates the differentiation of helper T cells through the selective activation of signaling by mTORC1 and mTORC2. Nat Immunol.

[7] Hay N, Sonenberg N (2004). Upstream and downstream of mTOR. Genes Dev.

[8] Weichhart T, Säemann MD (2009). The multiple facets of mTOR in immunity. Trends Immunol.

[9] Fruman DA (2004). Towards an understanding of isoform specificity in phosphoinositide 3-kinase signalling in lymphocytes. Biochem Soc Trans.

[10] Delgoffe GM, Kole TP, Zheng Y, Zarek PE, Matthews KL, Xiao B (2009). The mTOR kinase differentially regulates effector and regulatory T cell lineage commitment. Immunity.

[11] Sinclair LV, Finlay D, Feijoo C, Cornish GH, Gray A, Ager A (2008). Phosphatidylinositol-3-OH kinase and nutrient-sensing mTOR pathways control T lymphocyte trafficking. Nat Immunol.

[12] Wicker LS, Boltz RC Jr, Matt V, Nichols EA, Peterson LB, Sigal NH (1990). Suppression of B cell activation by cyclosporin A, FK506 and rapamycin. Eur J Immunol.

[13] Donahue AC, Fruman DA (2007). Distinct signaling mechanisms activate the target of rapamycin in response to different B-cell stimuli. Eur J Immunol.

[14] Säemann MD, Haidinger M, Hecking M, Hörl WH, Weichhart T (2009). The multifunctional role of mTOR in innate immunity: implications for transplant immunity. Am J Transplant.

[15] Thomson AW, Turnquist HR, Raimondi G (2009). Immunoregulatory functions of mTOR inhibition. Nat Rev Immunol.

[16] Aliyari Z, Alemi F, Brazvan B, Tayefi Nasrabadi H, Nozad Charoudeh H (2015). CD26+ Cord Blood Mononuclear Cells Significantly Produce B, T, and NK Cells. Iran J Immunol.

[17] Kobayashi H, Dubois S, Sato N, Sabzevari H, Sakai Y, Waldmann TA (2005). Role of trans-cellular IL-15 presentation in the activation of NK cell-mediated killing, which leads to enhanced tumor immunosurveillance. Blood.

[18] Cooper MA, Fehniger TA, Turner SC, Chen KS, Ghaheri BA, Ghayur T (2001). Human natural killer cells: a unique innate immunoregulatory role for the CD56(bright) subset. Blood.

[19] Shillingford JM, Murcia NS, Larson CH, Low SH, Hedgepeth R, Brown N (2006). The mTOR pathway is regulated by polycystin-1, and its inhibition reverses renal cystogenesis in polycystic kidney disease. Proc Natl Acad Sci U S A.

[20] Weimbs T (2007). Polycystic kidney disease and renal injury repair: common pathways, fluid flow, and the function of polycystin-1. Am J Physiol Renal Physiol.

[21] Khaziri N, Mohammadi M, Aliyari Z, Soleimani Rad J, Tayefi Nasrabadi H, Nozad Charoudeh H (2016). Cord Blood Mononuclear Cells Have a Potential to Produce NK Cells Using IL2Rg Cytokines. Adv Pharm Bull.

[22] Rochman Y, Spolski R, Leonard WJ (2009). New insights into the regulation of T cells by γc family cytokines. Nat Rev Immunol.

[23] Copley MR, Beer PA, Eaves CJ (2012). Hematopoietic stem cell heterogeneity takes center stage. Cell Stem Cell.

[24] Brazvan B, Farahzadi R, Mohammadi SM, Montazer Saheb S, Shanehbandi D, Schmied L (2016). Key Immune Cell Cytokines Affects the Telomere Activity of Cord Blood Cells In vitro. Adv Pharm Bull.

[25] Powell JD, Pollizzi KN, Heikamp EB, Horton MR (2012). Regulation of immune responses by mTOR. Annu Rev Immunol.

[26] Yang H, Wang X, Zhang Y, Liu H, Liao J, Shao K (2014). Modulation of TSC-mTOR signaling on immune cells in immunity and autoimmunity. J Cell Physiol.

[27] Contreras AG, Dormond O, Edelbauer M, Calzadilla K, Hoerning A, Pal S (2008). mTOR-understanding the clinical effects. Transplant Proc.

[28] Nourse J, Firpo E, Flanagan WM, Coats S, Polyak K, Lee MH (1994). Interleukin-2-mediated elimination of the p27Kip1 cyclin-dependent kinase inhibitor prevented by rapamycin. Nature.

